# Supplementary material for: PGE2 production at sites of tissue injury promotes an anti-inflammatory neutrophil phenotype and determines the outcome of inflammation resolution in vivo
Source: Sci Adv. 2018 Sep 5;4(9):eaar8320. doi: 10.1126/sciadv.aar8320 (PMC6124908; doi:10.1126/sciadv.aar8320)
Supplement: http://advances.sciencemag.org/cgi/content/full/4/9/eaar8320/DC1 [file supp_4_9_eaar8320__index.html]

Science Advances | Science Advances

## Supplementary Materials

**This PDF file includes:**

- Fig. S1. Reverse transcription PCR demonstrates successful exon deletion.
- Fig. S2. PGE2 drives resolution of inflammation in the absence of macrophages by 12 hpi and does not alter neutrophil apoptosis.
- Fig. S3. Amino acid positions 353 and 418 determine LOX functionality.
- Fig. S4. Genotyping method for *ptges* crispant.

Download PDF

**Files in this Data Supplement:**

- Adobe PDF - aar8320\_SM.pdf
