# Supplementary material for: PGE2 production at sites of tissue injury promotes an anti-inflammatory neutrophil phenotype and determines the outcome of inflammation resolution in vivo
Source: Sci Adv. 2018 Sep 5;4(9):eaar8320. doi: 10.1126/sciadv.aar8320 (PMC6124908; doi:10.1126/sciadv.aar8320)
Supplement: http://advances.sciencemag.org/cgi/content/full/4/9/eaar8320/DC1 [file aar8320_SM.pdf]

Science Advances
AAAS

advances.sciencemag.org/cgi/content/full/4/9/eaar8320/DC1

Supplementary Materials for

PGE_2 production at sites of tissue injury promotes an anti-inflammatory neutrophil phenotype and determines the outcome of inflammation resolution in vivo

Catherine A. Loynes, Jou A. Lee, Anne L. Robertson, Michael JG. Steel, Felix Ellett, Yi Feng, Bruce D. Levy, Moira K.B. Whyte, Stephen A. Renshaw*

*Corresponding author. Email: s.a.renshaw@sheffield.ac.uk

Published 5 September 2018, *Sci. Adv.* **4**, eaar8320 (2018)
DOI: 10.1126/sciadv.aar8320

This PDF file includes:

Fig. S1. Reverse transcription PCR demonstrates successful exon deletion.
Fig. S2. PGE_2 drives resolution of inflammation in the absence of macrophages by 12 hpi and does not alter neutrophil apoptosis.
Fig. S3. Amino acid positions 353 and 418 determine LOX functionality.
Fig. S4. Genotyping method for *ptges* crispant.

Supplementary Materials

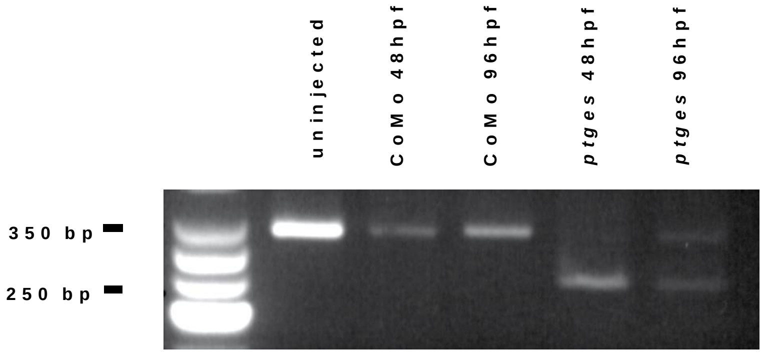

Fig. S1. Reverse transcription PCR demonstrates successful exon deletion. Gel electrophoresis showing almost complete knockdown of *ptges* at 48 and partial knockdown remaining at 96 hpf. A splice variant missing exon 2 in *ptges* morphants of 250bp compared to a WT product of 350bp is visible.

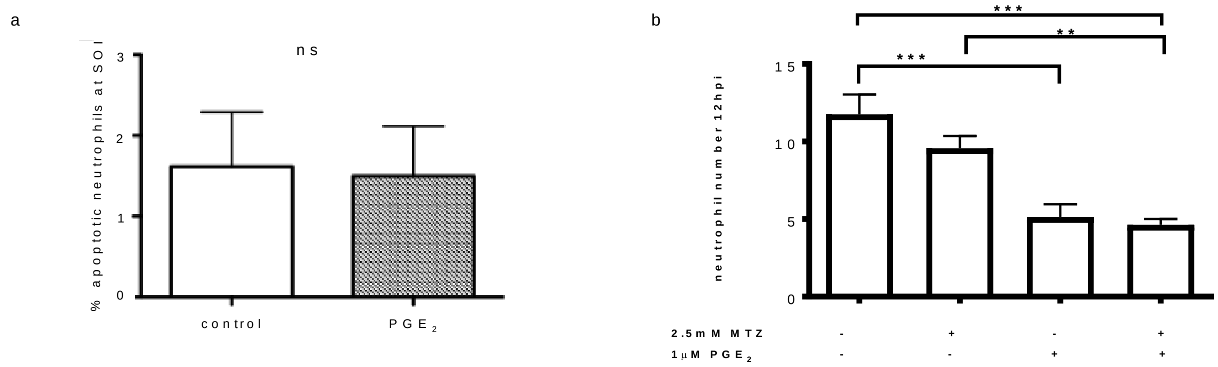

Fig. S2. PGE$_2$ drives resolution of inflammation in the absence of macrophages by 12 hpi and does not alter neutrophil apoptosis. (**A**) Graph showing PGE$_2$ does not affect neutrophil apoptosis during inflammation resolution. Larvae were injured, treated with PGE$_2$ and fixed at 12 hpi for TSA and TUNEL staining. The percentage of apoptotic neutrophils at the site of injury was not altered by the addition of PGE$_2$. n=36, from 2 experimental repeats. (**B**) Neutrophil counts 12 hpi in 3 dpf *Tg(mpx:EGFP)i114;Tg(cfms:Gal4)i186;Tg(UAS:nfsB-mCherry)i149* larvae treated with and without 2.5mM metronidazole and 1μM PGE$_2$. PGE$_2$, added at 8 hpi, can significantly drive inflammation resolution by 12 hpi, even when

macrophages are still being recruited to the site of injury in control larvae or in their complete absence when treated with metronidazole.

```
Medalox12     -HPDDGLIPIAIQLEQT--PGLDTPIFLPSDPPLAWLLAKMWVRHAEFQVFQLLSHLLRT   396
Zfalox12      -HPEKGLIPIAIQLEQK--PDKDTPVFLPSDPPLAWLLAKMWVRHAEFQVFQLLSHLLRT   371
Cfalox12      -HPESGLIPIAIQLEQN--PGKDTPIFLPNDPPLAWLLAKMWVRHAEFQVFQLLSHLLRT   370
Halox12       -EPNGKLQPMVIQIQPPNPSSPTPTLFLPSDPPLAWLLAKSWVRNSDFQLHEIQYHLLNT   364
Malox12       -DPGGKLLPMAIQIQPPNPSSPAPTLFLPSDPPLAWLLAKIWVRNSDFQLQELQFHLLNT   364
Malox15       -QPDGQLLPIAIQLELPKTGSTPPPIFTPLDPPMDWLLAKCWVRSSDLQLHELQAHLLRG   365
Ralox12       -QPDGKLMPMVIQLHLPKIGSSPPPLFLPTDPPMVWLLAKCWVRSSDLQVHELNSHLLRG   365
Ralox15       QQPSTVGLVICLELHLPKIGSSPPPLFLPTDPPMVWLLAKCWVRSSDFQVHELNSHLLRG   495
Halox15       -QPDGKLLPMVIQLQLPRTGSPPPPLFLPTDPPMAWLLAKCWVRSSDFQLHELQSHLLRG   364
Palox15       -QPDGKLLPMVIQLQLPHEGSPLPPLFLPTDPPMVWLLAKCWVRSSDFQLHELHSHLLRG   365
Malox8        SSGSGPLLPIAIQLKQT--PGPDNPIFLPSDDTWDWLLAKTWVRNSEFYIHEAVTHLLHA   378
Medalox15     MNPEGKLMPLAIQLNQK--ASAENPIFLPTDPEKDWLLAKLFFKSADLLECEVVHHLLIT   388
                       : :::.     .    :* * *    ***** :.: :::   :   ***

Medalox12     HLVVEVFCVATLRQLPAVHPIYKLLAPHQRYTLEINCRGRTQLLSETGIFKRVVSTGGEG   456
Zfalox12      HLMVEVICVATLRQLPAVHPIYKLLTPHLRYTLEINCRGRTQLLSPEGIFKRVVSTGGEG   431
Cfalox12      HLVVEVICVSTLRHLPAVHPIYKLLTPHLKYTLEINCRGRTQLISPEGIFKRVVSTGGTG   430
Halox12       HLVAEVIAVATMRCLPGLHPIFKFLIPHIRYTMEINTRARTQLISDGGIFDKAVSTGGGG   424
Malox12       HLVAEVIAVATMRCLPGLHPIFKLLVPHIRYTMEINTRSRTQLISDGGIFDQVVSTGGGG   424
Malox15       HLVAEVFAVATMRCLPSVHPVFKLLVPHLLYTMEINVRARSDLISERGFFDKVMSTGGGG   425
Ralox12       HLMAEVFTVATMRCLPSIHPVFKLIVPHLRYTLEINVRARNGLVSDFGIFDQIMSTGGGG   425
Ralox15       HLMAEVFTVATMRCLPSIHPVFKLIVPHLRYTLEINVRARNGLVSDFGIFDQIMSTGGGG   555
Halox15       HLMAEVIVVATMRCLPSIHPIFKLIIPHLRYTLEINVRARTGLVSDMGIFDQIMSTGGGG   424
Palox15       HLMAEVIAVATMRCLPSIHPIFKLLIPHFRYTMEINVRARNGLVSDLGIFDQVVSTGGGG   425
Malox8        HLIPEVFALATLRQLPRCHPLFKLLIPHIRYTLHINTLARELLVAPGKLIDKSTGLGTGG   438
Medalox15     HFLSEVFAVATLRCFPTIHPLHKLLIPHFRFTLHINIMGREALLGPDGALC-ASSFGLEG   447
              *:: **: ::*:* :*  **:.*:: **  :*:.**  .*  *:.    :    . *  *
```

Fig. S3. Amino acid positions 353 and 418 determine LOX functionality. Alignments of mammalian and fish 12- and 15-LO proteins. A Phenylalanine (F) at position 353 (magenta box) in reference to rabbit 12-LO, and a bulky amino acid Valine (V) at position 418 (green box) dictates preferential oxygenation at carbon 15, indicating zebrafish 12-LO functions as a 15-LO lipoxygenase.

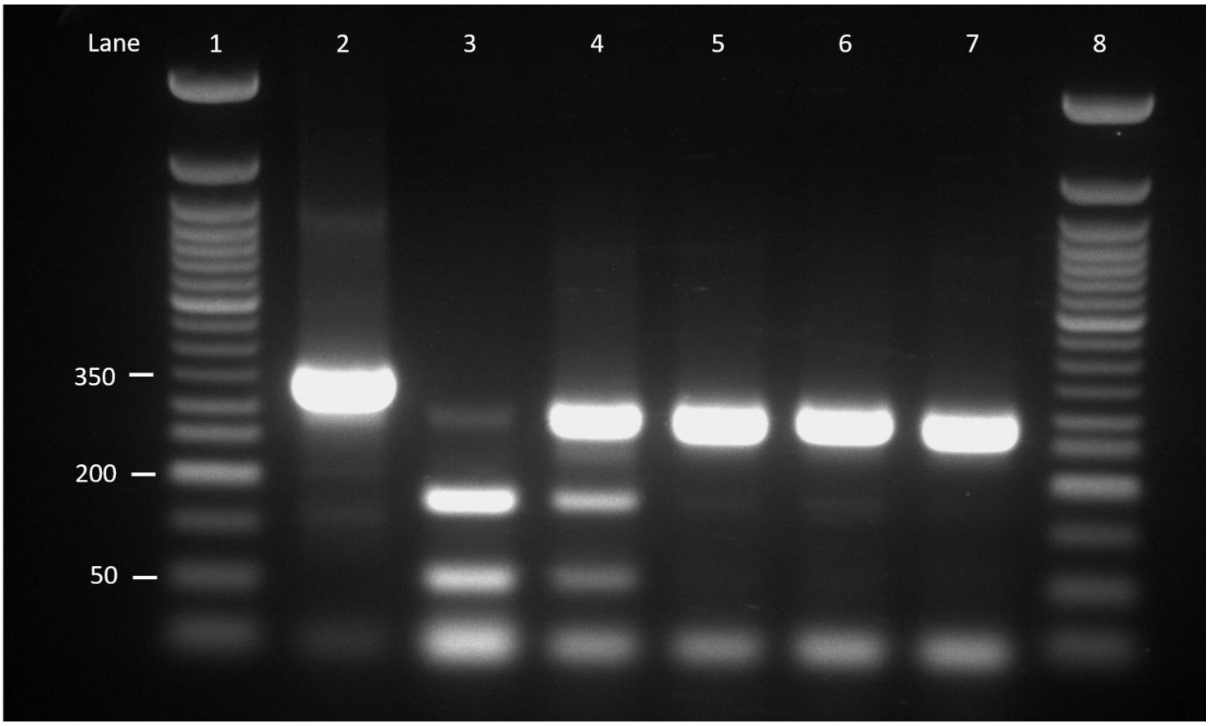

Fig. S4. Genotyping method for *ptges* crispant. Electrophoresis gel shows different genotypes from *ptges* CRISPR injection. Lane 1: NEB 50bp ladder. Lane 2: undigested PCR product 345bp. Lane 3: fully cut WT 184bp, 109bp and 52bp. Lane 4: Heterozygous CRISPant with 293bp band, 184bp, 109bp and 52bp. Lane 5-7: Homozygous CRISPants with 293bp and 52bp bands. Very weak 184bp and 109bp bands can be see, indicating a small amount of WT *ptges* is still present, allowing for normal development in these almost complete knockdown CRISPant larvae. Lane 8: NEB 50bp ladder.
